# Giant Necrotic Axillary Metastasis From Pancreatic Neuroendocrine Carcinoma Treated With Palliative Resection and Breast Reconstruction: A Case Report

**DOI:** 10.7759/cureus.113112

**Published:** 2026-07-21

**Authors:** Benjamin Moya Leal, Martín Rodríguez-Garza, Cynthia L Nava Palomo, Nancy G Guzman, Mario Leal-Maldonado, Jorge A Garcia Garza, Ricardo Valdés Espinosa

**Affiliations:** 1 General Surgery, Instituto de Seguridad y Servicios Sociales de los Trabajadores del Estado (ISSSTE) Hospital Regional de Monterrey, Monterrey, MEX; 2 General Surgery, Instituto de Seguridad y Servicios Sociales de los Trabajadores del Estado (ISSSTE), Monterrey, MEX; 3 General Surgery, Instituto de Seguridad y Servicios Sociales de los Trabajadores del Estado (ISSSTE) Clínica Hospital Constitución, Monterrey, MEX; 4 General Surgery, University of Monterrey, Monterrey, MEX; 5 General Surgery, Secretaria de Salud, Saltillo, MEX; 6 General Surgery, Hospital General y de Alta Especialidad del Instituto de Seguridad y Servicios Sociales de los Trabajadores del Estado (ISSSTE), Saltillo, MEX

**Keywords:** axillary metastasis, case report, giant mass, neuroendocrine tumor, palliative surgery, pancreatic neuroendocrine tumor, reconstructive surgery

## Abstract

Neuroendocrine tumors of the pancreas are rare and can present with atypical metastatic patterns. Axillary involvement is extremely uncommon and requires a diagnosis of exclusion. Furthermore, it can be a therapeutic challenge.

We present the case of a 78-year-old woman with a medical history of arterial hypertension, type 2 diabetes mellitus, and Parkinson’s disease. She was diagnosed with pancreatic carcinoma with metastatic disease to axillary nodes. The patient developed a massive necrotic axillary tumor, measuring approximately 30 × 30 cm clinically, also associated with erythema, pain, exudate, and local infection. Laboratory tests showed severe anemia (Hb 7.4 g/dL), neutrophilia (95.1%), hyponatremia (Na 132 mmol/L), and hypokalemia (K 2.3 mmol/L). Imaging (contrast-enhanced CT and angio-CT) revealed a well-defined mass displacing the pectoral, deltoid, and serratus muscles; partially compressing the brachial artery with distal recanalization; and measuring approximately 25 cm x 20 cm. Three months after the first diagnosis, the patient underwent a palliative surgery including a block resection of the axillary tumor measuring 26 × 21 × 8 cm and weighing 3000 g. Histopathology results showed a poorly differentiated metastatic neuroendocrine carcinoma with rhabdoid features, extensive necrosis throughout the whole tumor, and positive margins. Immunohistochemistry was also performed and revealed focal enolase positivity, while CD56, p53, and chromogranin were negative.

Pancreatic neuroendocrine tumors usually metastasize to the liver, peritoneum, or lymph nodes. Axillary metastasis is extremely rare and has been documented in only a few cases. The presence of a large, necrotic, infected tumor in this location significantly impacts quality of life. Surgical resection, although not curative, may provide substantial pain relief, control local complications, reduce infectious sequelae, and improve quality of life. This particular case highlights an uncommon metastatic site of pancreatic neuroendocrine carcinoma. Awareness of atypical metastatic patterns is essential for more precocious management, and surgical resection can provide substantial palliative benefit in selected patients.

## Introduction

Pancreatic neuroendocrine tumors are a heterogeneous group of tumors with complicated treatment options that depend on pathological grading, clinical staging, and the presence of symptoms related to hormonal secretion [1,2]. Pancreatic neuroendocrine neoplasms comprise a heterogeneous group of tumors with variable biological behavior, ranging from indolent lesions to highly aggressive, poorly differentiated carcinomas. Common metastatic sites include the liver, lungs, and peritoneum. Early diagnosis remains essential, as prognosis is strongly associated with disease stage at presentation [3].

Although lymphatic spread to axillary nodes can occasionally occur in intra-abdominal malignancies, direct soft tissue metastasis forming a massive axillary lesion is exceedingly rare, especially from pancreatic neuroendocrine carcinomas [3]. Only isolated case reports describe axillary involvement from pancreatic origin, most of them adenocarcinomas, with even fewer cases reported in neuroendocrine tumors [3,4]. These atypical presentations pose significant diagnostic challenges, as they may mimic primary soft tissue sarcomas, breast carcinoma, or infectious processes. Furthermore, the management of giant symptomatic metastases in frail elderly patients with disseminated disease requires a careful balance between surgical risk and potential palliative benefit, particularly when reconstructive surgery is needed to restore function and body contour.

We report the case of a 78-year-old woman who developed a massive, necrotic, and infected axillary metastasis from a pancreatic neuroendocrine carcinoma. This case highlights the diagnostic difficulties, the surgical complexity (including reconstructive aspects), and the palliative benefit of resection in carefully selected patients with advanced disease.

## Case presentation

A 78-year-old woman with a medical history of arterial hypertension, type 2 diabetes mellitus, and Parkinson’s disease was diagnosed with pancreatic neuroendocrine carcinoma of the tail with synchronous metastatic disease (liver and axillary lymphadenopathy) after evaluation for abdominal pain and weight loss. She initially received systemic treatment with a poor response.

Three months after the initial diagnosis, she presented with rapid enlargement of a right axillary mass accompanied by severe pain, erythema, purulent exudate, foul odor, and progressive limitation of right upper limb mobility. On physical examination, a giant mass measuring approximately 30 × 30 cm was observed, with signs of local infection and abscess formation.

**Figure 1:**
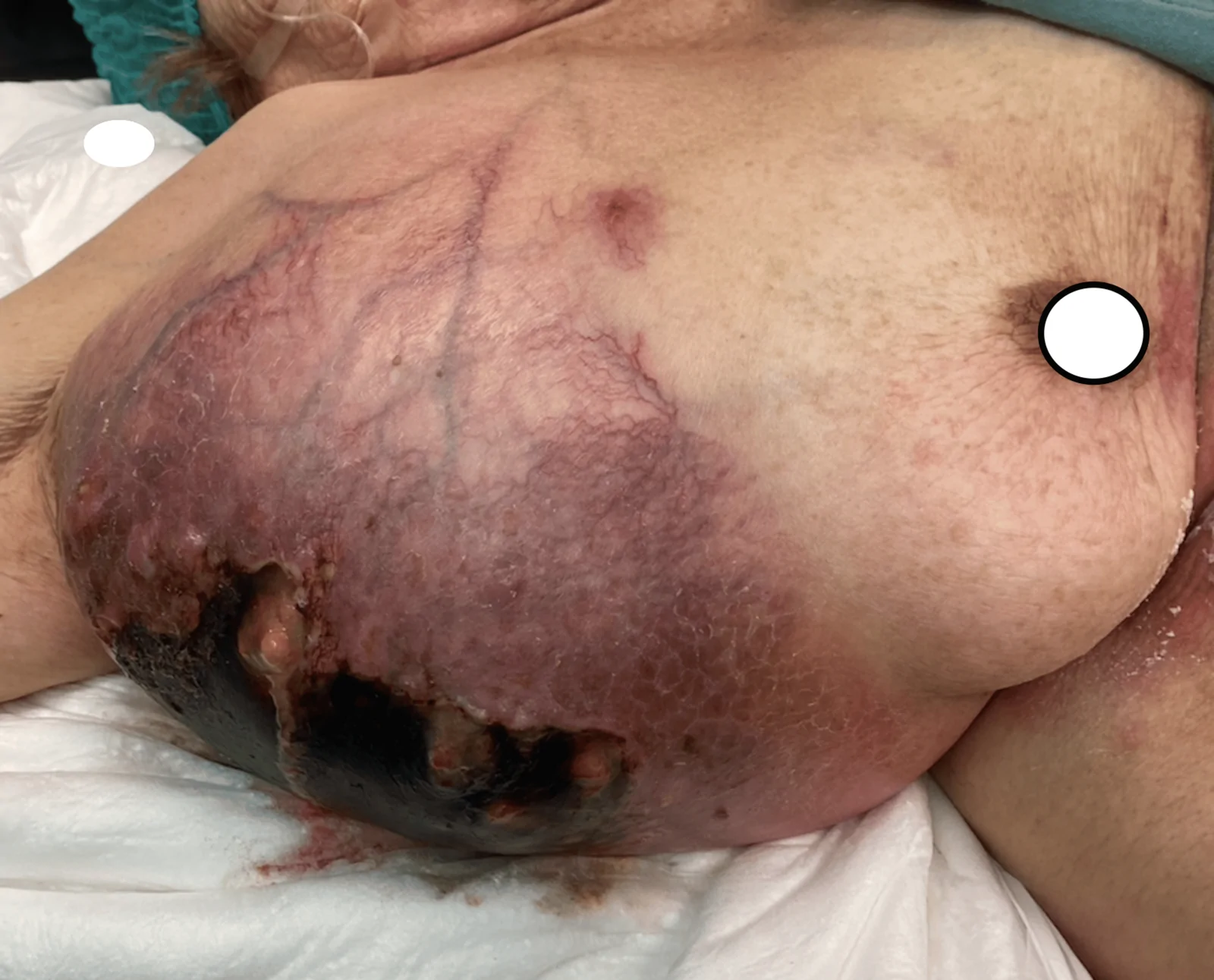
Preoperative clinical appearance of the giant right axillary metastasis The preoperative photograph demonstrates a giant right axillary metastatic mass measuring approximately 30 × 30 cm. The lesion shows marked skin tension, extensive superficial venous engorgement, areas of full-thickness skin necrosis, multiple abscess cavities with purulent drainage, hemorrhagic crusts, and surrounding erythema, consistent with advanced local tumor progression complicated by secondary infection.

Laboratory tests revealed severe anemia (Hb 7.4 g/dL), marked neutrophilia (95.1%), hyponatremia (Na 132 mmol/L), and hypokalemia (K 2.3 mmol/L). Contrast-enhanced CT and angio-CT demonstrated a heterogeneous, well-defined mass measuring approximately 25 × 20 cm, with central necrosis and abscess formation. The mass displaced the pectoralis major, deltoid, and serratus anterior muscles and caused partial compression of the brachial artery with distal recanalization through collateral circulation (Figure 1).

**Figure 2:**
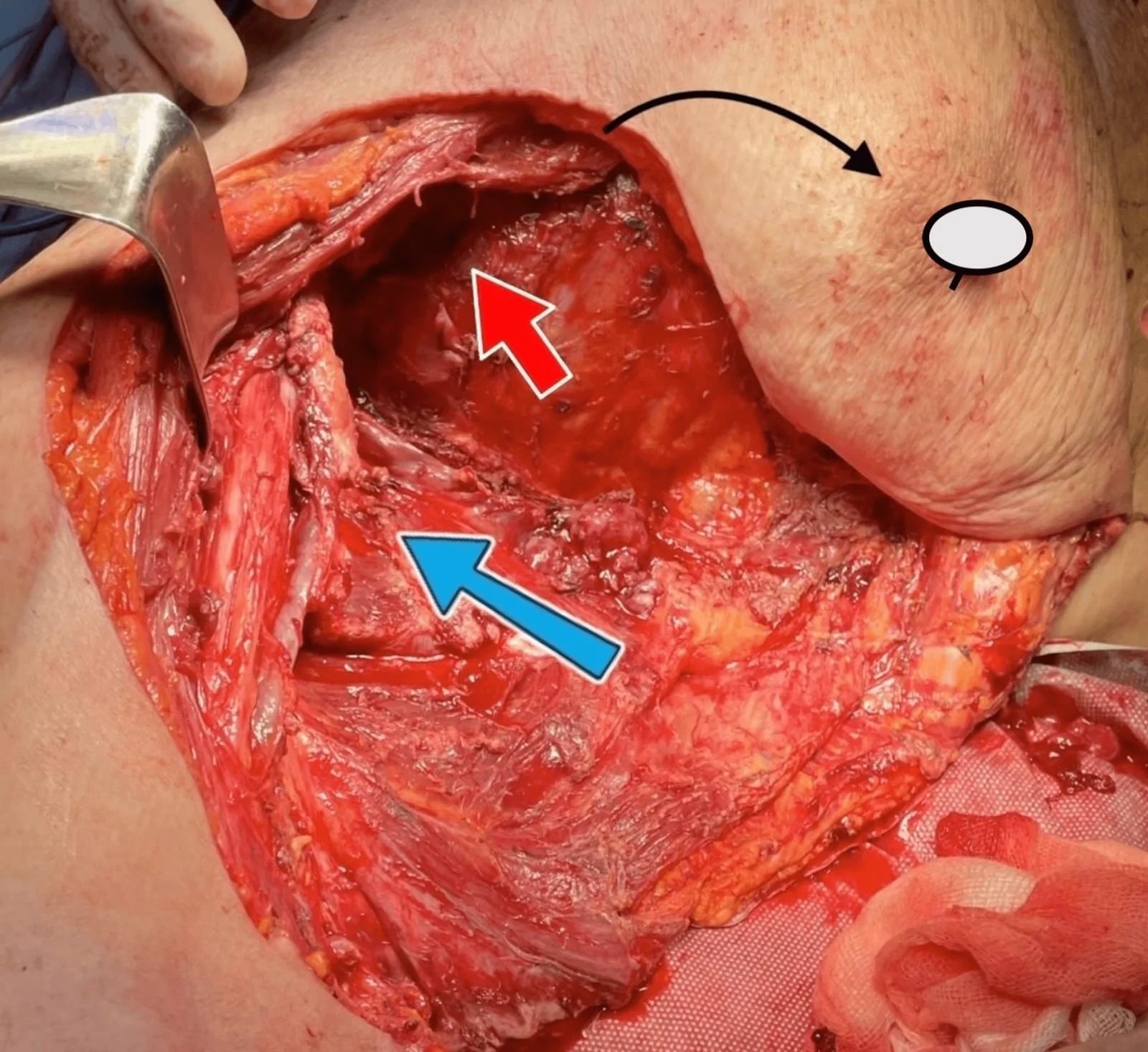
Intraoperative view after en-bloc resection of the giant axillary metastasis The intraoperative photograph demonstrates the S-shaped incision used to expose the axillary region following tumor excision. The operative field shows preservation of the pectoralis major muscle (red arrow), the axillary neurovascular bundle (blue arrow), and the remaining breast tissue with an intact nipple-areola complex (black arrow). These preserved structures allowed immediate reconstruction of the surgical defect after complete en-bloc resection of the metastatic mass.

**Figure 3:**
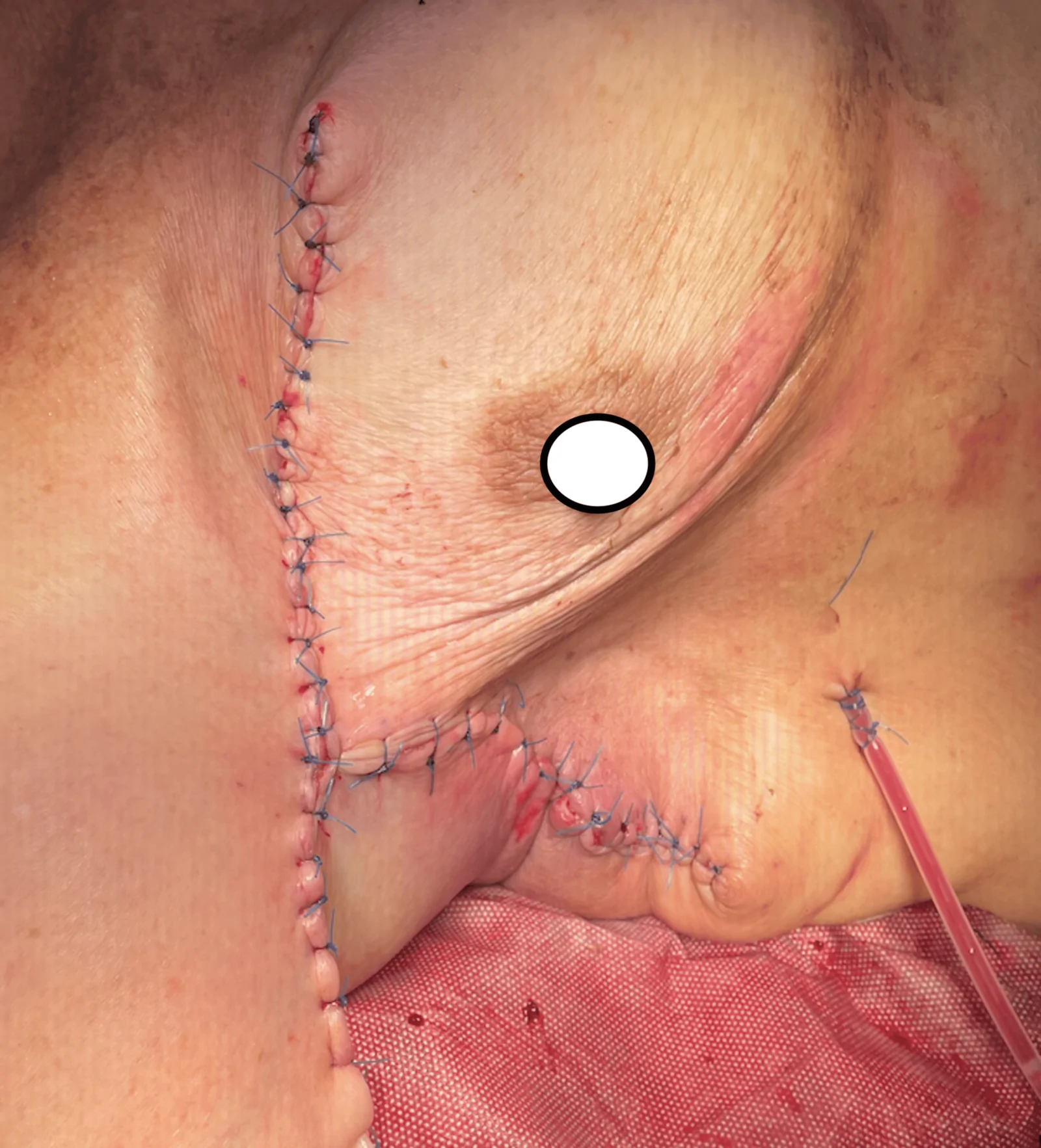
Immediate postoperative reconstruction after en-bloc resection of the tumor The clinical photograph obtained immediately after tumor excision shows successful primary reconstruction of the axillary defect. Preservation of the pectoralis major muscle, axillary vessels, and nipple-areola complex allowed reconstruction using the remaining breast tissue, achieving satisfactory soft tissue coverage, no ischemic borders, and breast contour following resection of the 3-kg metastatic tumor.

A curvilinear S-shaped incision was performed along the inferior clavicular border extending toward the axillary region. Careful dissection was carried out with preservation of major neurovascular structures, including the right axillary vessels (Figure 2). The tumor was identified as a giant necrotic mass occupying the axillary region, measuring approximately 30 × 30 cm, with areas of abscess formation, foul odor, extensive inflammatory changes, and soft tissue involvement. En bloc resection of the lesion was successfully achieved while preserving the pectoralis major muscle, as evidenced intraoperatively. Following tumor excision, layered closure was performed. Reconstruction of the remaining breast glandular tissue was carried out with preservation of the nipple-areola complex to optimize chest wall coverage and postoperative contour (Figure 3). The resected specimen measured 26 × 21 × 8 cm and weighed approximately 3000 g. Postoperatively, the patient remained hemodynamically stable, with significant improvement in local pain, inflammatory changes, progressive recovery of mobility in the upper limb and infection-related symptoms. 

## Discussion

Pancreatic neuroendocrine neoplasms are uncommon tumors characterized by a broad spectrum of biological behavior, ranging from indolent lesions to highly aggressive, poorly differentiated carcinomas with early metastatic potential [1-4]. Metastatic disease is frequently present at diagnosis, particularly in high-grade tumors, with regional lymph nodes, liver, lungs, and bone representing the most common sites of dissemination [1-4]. In contrast, axillary involvement is exceptionally uncommon and has rarely been reported in the literature. Most published reports of axillary metastases originate from breast, gastrointestinal, or pulmonary malignancies, whereas pancreatic tumors seldom metastasize to this location [5-9]. Furthermore, among pancreatic neoplasms, the majority of reported axillary metastases derive from pancreatic adenocarcinoma rather than neuroendocrine carcinoma, making the present case particularly unusual [8-10].

The pathophysiological mechanisms responsible for axillary dissemination from pancreatic tumors remain poorly understood. Proposed explanations include aberrant lymphatic drainage pathways, hematogenous spread, and dissemination through collateral lymphatic channels secondary to advanced disease burden [6,9]. The rarity of this metastatic pattern often leads clinicians to consider alternative diagnoses such as primary breast carcinoma, soft tissue sarcoma, lymphoma, or infectious processes before establishing the correct diagnosis [2,6,9]. In our patient, the presence of a rapidly enlarging necrotic mass associated with abscess formation and severe inflammatory changes further complicated the diagnostic evaluation.

Another distinctive aspect of this case was the rhabdoid phenotype identified on histopathological examination. Microscopically, the lesion demonstrated nests of poorly differentiated malignant cells with abundant eosinophilic cytoplasm, eccentric hyperchromatic nuclei, marked pleomorphism, and extensive soft tissue infiltration. Rhabdoid differentiation has been associated with highly aggressive biological behavior in multiple malignancies and is generally considered a marker of poor prognosis [4]. Immunohistochemical analysis demonstrated focal positivity for neuron-specific enolase (NSE), whereas CD56, chromogranin, and p53 were negative. Loss of conventional neuroendocrine markers has been described in poorly differentiated neuroendocrine carcinomas and may create diagnostic challenges, particularly in metastatic lesions with unusual morphology [4]. The presence of a rhabdoid phenotype likely reflects tumor dedifferentiation and may partially explain the aggressive local progression observed in this patient.

The remarkable size of the lesion also deserves consideration. The tumor measured approximately 30 × 30 cm clinically and 26 × 21 × 8 cm following resection, with a total weight of approximately 3000 g. To our knowledge, this represents one of the largest reported axillary metastatic lesions arising from a pancreatic neuroendocrine carcinoma. Beyond its oncologic significance, the lesion produced substantial morbidity, including severe pain, local infection, purulent drainage, foul odor, skin compromise, and progressive limitation of upper extremity mobility. These symptoms significantly impaired the patient's quality of life and ultimately became the primary indication for surgical intervention.

The role of palliative surgery in advanced malignancy has evolved considerably over recent decades. Although curative treatment remains the ideal goal whenever feasible, surgical intervention may be justified in patients experiencing significant local symptoms such as pain, ulceration, bleeding, infection, malodor, obstruction, or functional impairment [5,11,12]. In such situations, the objective shifts from prolongation of survival to symptom control and preservation of quality of life. Several studies have demonstrated that appropriately selected palliative surgical procedures can provide substantial symptomatic improvement and enhance patient comfort despite the presence of incurable disease [11,12].

Contemporary evidence further suggests that quality of life should be considered a primary endpoint when evaluating therapeutic interventions in patients with advanced malignancies. A recent meta-analysis demonstrated that palliative care interventions significantly improve symptom burden, emotional well-being, and overall quality of life compared with standard oncologic care alone [5,13]. These findings support a patient-centered approach in which treatment decisions are guided not only by survival expectations but also by symptom control, preservation of function, and maintenance of dignity throughout the disease course.

In the present case, systemic progression precluded curative intent; however, the giant necrotic axillary mass had become the dominant source of morbidity. En bloc resection allowed effective drainage and removal of infected tissue, reduction of tumor burden, elimination of malodor, improvement in local wound conditions, and restoration of upper extremity function. Following surgery, the patient experienced significant relief of pain and inflammatory symptoms. Although positive margins were identified histologically, the primary objective of palliation was achieved. These findings reinforce the concept that surgical management should not be dismissed solely because metastatic disease is present, particularly when local symptoms substantially compromise quality of life [5,11,12].

The reconstructive component of the procedure represented another important aspect of treatment. Resection of giant axillary tumors frequently generates extensive soft tissue defects that may require complex reconstructive strategies. In our patient, preservation of the pectoralis major muscle, axillary vascular structures, and nipple-areola complex allowed immediate reconstruction using the remaining breast glandular tissue. This approach provided adequate soft tissue coverage while preserving chest wall contour and minimizing additional operative morbidity. In elderly patients with advanced oncologic disease, reconstructive planning should prioritize reliable wound closure, rapid recovery, preservation of function, and acceptable aesthetic outcomes rather than extensive reconstructive procedures [5].

Finally, this case highlights the importance of multidisciplinary management involving surgical oncology, pathology, internal medicine, radiology, and reconstructive surgery. Recognition of atypical metastatic patterns is essential because unusual presentations may delay diagnosis and treatment. Although metastatic pancreatic neuroendocrine carcinoma remains associated with poor prognosis, carefully selected palliative surgical interventions can offer meaningful clinical benefit by controlling symptoms, reducing complications, and improving quality of life [11-13].

The principal limitations of this report include its single-patient design, the absence of Ki-67 proliferation index data, which was unavailable, lack of molecular characterization, and limited postoperative follow-up. Nevertheless, the unusual metastatic location, giant tumor size, rhabdoid histology, and reconstructive considerations contribute valuable information to the limited literature available regarding axillary metastases from pancreatic neuroendocrine carcinoma.

## Conclusions

This case illustrates a rare giant axillary metastasis from pancreatic neuroendocrine carcinoma with rhabdoid features. Tumors of pancreatic origin should be considered in the differential diagnosis of atypical or rapidly growing axillary masses. Although not curative, palliative en bloc resection with reconstruction provided effective symptom control, infection resolution, pain relief, and improved quality of life in this patient with advanced disease. Early recognition of unusual metastatic sites and a multidisciplinary approach are essential for optimal palliative management of pancreatic neuroendocrine carcinoma.
